# Risk factors for persistent tomographic abnormalities at 6 months of follow-up in a cohort of hospitalized patients with moderate and severe COVID-19 living at high altitude

**DOI:** 10.3389/fmed.2023.1110535

**Published:** 2023-02-08

**Authors:** Emily Rincon-Alvarez, Mauricio Gonzalez-Garcia, Abraham Ali-Munive, Alejandro Casas, Nadia Juliana Proaños, Luis Fernando Giraldo-Cadavid, Angelica Moreno, Carolina Pérez, Wendy Rubiano, Mary Cogollo, Patricia Parada-Tovar, Carlos A. Torres-Duque

**Affiliations:** ^1^Fundación Neumológica Colombiana, Bogotá, Colombia; ^2^Fundación Cardioinfantil, Bogotá, Colombia; ^3^Facultad de Medicina, Universidad de La Sabana, Chía, Colombia

**Keywords:** COVID-19, altitude, six-minute walk test, respiratory function tests, computed tomography, dyspnea

## Abstract

**Introduction:**

After COVID-19, functional and tomographic lung alterations may occur, but there are no studies at high altitude where, due to lower barometric pressure, there are lower levels of arterial oxygen pressure and saturation in both normal subjects and patients with respiratory disease. In this study, we evaluated the computed tomographic (CT), clinical, and functional involvement at 3 and 6 months post-hospitalization in survivors with moderate-severe COVID-19, as well the risk factors associated with abnormal lung computed tomography (ALCT) at 6 months of follow-up.

**Materials and methods:**

Prospective cohort, after hospitalization for COVID-19, of patients older than 18 years residing at high altitude. Follow-up at 3 and 6 months with lung CT, spirometry, diffusing capacity of the lung for carbon monoxide (DLCO), six-minute walk test (6MWT), and oxygen saturation (SpO_2_). Comparisons between ALCT and normal lung computed tomography (NLCT) groups with X^2^ and Mann–Whitney U test, and paired test for changes between 3 and 6 months. A multivariate analysis was performed to evaluate the variables associated with ALCT at 6-month follow-up.

**Results:**

We included 158 patients, 22.2% hospitalized in intensive care unit (ICU), 92.4% with typical COVID CT scan (peripheral, bilateral, or multifocal ground glass, with or without consolidation or findings of organizing pneumonia), and median hospitalization of 7 days. At 6 months, 53 patients (33.5%) had ALCT. There were no differences between ALCT and NLCT groups in symptoms or comorbidities on admission. ALCT patients were older and more frequently men, smokers and hospitalized in ICU. At 3 months, ALCT patients had more frequently a reduced forced vital capacity (< 80%), and lower meters walked (6MWT) and SpO_2_. At 6 months, all patients improved lung function with no differences between groups, but there were more dyspnea and lower exercise SpO_2_ in ALCT group. The variables associated with ALCT at 6 months were age, sex, ICU stay, and typical CT scan.

**Conclusion:**

At 6-month follow-up, 33.5% of patients with moderate and severe COVID had ALCT. These patients had more dyspnea and lower SpO_2_ in exercise. Regardless of the persistence of tomographic abnormalities, lung function and 6MWT improved. We identified the variables associated with ALCT.

## Introduction

Severe acute respiratory syndrome coronavirus 2 (SARS-CoV-2), which causes coronavirus disease 2019 (COVID-19), can compromise the lower respiratory tract and cause pneumonia (1). COVID-19 was declared a pandemic on 6 March 2020, and has caused 606,795,204 reported cases and 6,507,435 deaths in the world (2). In Colombia, as of 1st September 2022, 6,302,809 cases and 142,259 deaths have been reported (2).

A significant proportion of patients with COVID-19, particularly the more severe cases, can develop long-term functional and radiographic abnormalities (3). These findings have been previously described in patients with coronavirus infections that developed severe acute respiratory syndrome (SARS-CoV) (4, 5). In survivors of middle east respiratory syndrome coronavirus (MERS-CoV), at 6 weeks of follow-up radiographic abnormalities were found including pulmonary fibrosis, the presence of ground glass, and pleural thickening (6, 7). Respiratory sequelae after recovery from COVID-19 infection have not been fully reported and have become a cause for concern, not only because it is today one of the main reasons for consultation, but also because of the injuries that can be found in the long term.

Some studies, with a follow-up of 3–6 months, have reported the presence of post-infection symptoms such as fatigue, muscle weakness, anxiety, and, in patients with a more critical condition, alterations in the diffusing capacity of the lung for carbon monoxide (DLCO) and abnormalities in the chest tomography [computed tomographic (CT) scan] (8). In a study with a follow-up of up to 1 year, improvement in forced vital capacity (FVC) and the six-minute walk test (6MWT) has been reported, although with alterations in DLCO and persistence of abnormalities in the CT scan (9).

At altitude, due to lower barometric pressure and lower inspired pressure of oxygen, there are lower levels of arterial oxygen pressure and saturation in both normal subjects and patients with respiratory disease (10, 11). More than 80 million people in the world live 2,500 m above sea level, mainly in Latin America and the Andean region (12). So far, some of the effects of the altitude on the severity of the acute presentation of COVID-19 disease are known, given by lower oxygenation indices upon admission to the intensive care unit (ICU) and the requirement for invasive mechanical ventilation (13), however, the medium-term effects of this disease are not known.

Our objective is to describe respiratory symptoms, lung function, and chest CT findings at 3 and 6 months after discharge of hospitalization for COVID-19-associated pneumonia in a population of patients older than 18 years living in a high-altitude city (Bogotá). In addition, to describe the risk factors that were associated with abnormal lung computed tomography (ALCT) at 6 months of follow-up.

## Materials and methods

Design and participants: a prospective cohort study with patients older than 18 years who required hospitalization due to a diagnosis of COVID-19 confirmed by PCR of nasopharyngeal secretion and lower respiratory tract involvement by clinical findings and CT. The participants consulted the emergency and outpatient services of the Fundación Cardioinfantil and Fundación Neumológica Colombiana between August 2020 and May 2021. All had to be residents of Bogotá, a city located 2,640 m above sea level, and complete an outpatient follow-up of up to 6 months.

Patients who died during the hospital stay and those with interstitial abnormalities on CT scan before COVID-19 were excluded. All subjects included signed informed consent and the study was approved by the Ethics Committee of the Fundación Neumológica Colombiana (approval number 202007-25702).

### Procedures

At hospital admission, sociodemographic variables, respiratory symptoms (cough, dyspnea, and chest pain), smoking habit, comorbidities, blood count, D-dimer, lactate dehydrogenase (LDH), ferritin, electrolytes, and arterial blood gases (ABG) were recorded. Dyspnea was assessed by modified medical research council (mMRC) score. In phase II (follow-up at 3 and 6 months after hospital discharge), clinical evaluation and respiratory function tests were performed, including spirometry, DLCO, ABG, 6MWT, and oxygen saturation (SpO_2_). CT scan was performed at admission and at 3-month follow-up in all patients, and at 6-month follow-up in those with ALCT at 3-month follow-up.

Pulmonary function tests were performed in a V-MAX Encore (CareFusion, Yorba Linda, CA, USA) in the pulmonary function laboratory of the Fundación Neumológica Colombiana according to the recommendations of the American Thoracic Society (ATS) and the European Respiratory Society (ERS), and Crapo reference equations were used (14–16). The 6MWT was performed according to ATS and ERS recommendations (17).

The CT scan was performed according to the technical recommendations of the American College of Radiology (18) in a Somaton Definition Edge equipment (Siemens). The interpretation was performed by a certified radiologist with chest experience as recommended by the Radiological Society of North America (RSNA) (19). The CT findings were classified into (1) Typical appearance: peripheral, bilateral, ground glass opacity (GGO) with or without consolidation or visible intralobular lines (“crazy-paving”); multifocal GGO of rounded morphology with or without consolidation or visible intralobular lines (“crazy-paving”); reverse halo sign or other findings of organizing pneumonia. (2) Indeterminate appearance: absence of typical features and presence of multifocal, diffuse, perihilar, or unilateral GGO with or without consolidation lacking a specific distribution and are non-rounded or non-peripheral; few very small GGO with a non-rounded and non-peripheral distribution. (3) Atypical appearance: absence of typical or indeterminate features and presence of: isolated lobar or segmental consolidation without GGO; discrete small nodules (centrilobular, “tree-in-bud”); lung cavitation; smooth interlobular septal thickening with pleural effusion. (4) Negative for pneumonia: no CT features to suggest pneumonia (19).

Abnormal lung computed tomography was defined as the persistence of pulmonary infiltrates in the CT scan at follow-up at 6 months, and normal lung computed tomography (NLCT) as the absence of infiltrates on the CT scan. All information was collected by REDCap software to minimize missing entries and allow data validation.

### Statistical analysis

The qualitative variables were described in relative and absolute frequencies, and the quantitative variables in measures of central tendency and dispersion according to the assumption of normality. For the comparison between qualitative variables between the ALCT and NLCT groups in the follow-up at 3 and 6 months, the *X*^2^ test or Fisher’s exact test was used. For non-parametric quantitative variables, the Mann–Whitney U test for independent samples was used. For comparisons between quantitative variables in the follow-up at 3 and 6 months, the paired Mann–Whitney U test was used.

A multivariable logistic regression model was performed to determine the variables associated with the persistence of tomographic abnormalities at 6 months of follow-up. Variables with a *p*-value < 0.25 in the initial bivariate analysis were included in the multivariate model. The model was evaluated in terms of the AUROC curve. The goodness of fit was evaluated using the Hosmer–Lemeshow test. For the analyses, the Stata 16 and R studio statistical programs were used, the tests were two-tailed, and a value of *p* < 0.05 was considered statistically significant.

## Results

### Demographics and characteristics at admission

A total of 158 patients were included, 57.6% men, with a median age of 55 years. At the six-month follow-up, 53 patients (33.5%) had ALCT. At admission, 26.0% of the subjects had a history of smoking and the main comorbidities were arterial hypertension (32.3%), obesity (29.8%), and diabetes (12.0%). Cough was the main symptom (77.2%), followed by dyspnea in 64.6% (Table 1).

**TABLE 1:** Baseline characteristics of the study population.

	Total *N* = 158	NLCT *N* = 105	ALCT *N* = 53	*p*
Age, years	55.0 (46.0–62.0)	51.0 (41.0–60.0)	59.0 (52.0–65.0)	< 0.001
Male sex	91 (57.6)	53 (50.5)	38 (71.7)	0.011
BMI, kg/m^2^	26.5 (24.1–30.3)	26.7 (24.6–31.6)	26.0 (23.8–30.2)	0.287
Smoker/former smoking	41 (26.0)	21 (20.0)	20 (37.7)	0.016
Obesity	47 (29.8)	33 (31.4)	14 (26.4)	0.515
Asthma	7 (4.4)	6 (5.7)	1 (1.9)	0.270
COPD	4 (2.5)	2 (1.9)	2 (3.8)	0.480
Hypertension	51 (32.3)	31 (29.5)	20 (37.7)	0.297
Type 2 diabetes	19 (12.0)	13 (12.4)	6 (11.3)	0.847
CKD	3 (1.9)	2 (1.9)	1 (1.9)	0.994
Chest pain	30 (19.0)	23 (21.9)	7 (13.2)	0.188
Cough	122 (77.2)	80 (76.2)	42 (79.3)	0.666
mMRC 0	56 (35.4)	36 (34.3)	20 (37.7)	0.669
mMRC ≥ 1	102 (64.6)	69 (65.7)	33 (62.3)	
LDH, U/L	398 (304–497)	388 (291–497)	428 (327–496)	0.157
D dimer, mg/L	1.0 (0.6–1.6)	0.9 (0.6–1.3)	1.4 (0.7–2.0)	0.017
Ferritin ng/ml	801 (402–1,781)	691 (359–1,529)	1,005 (564–2,351)	0.022
**Chest CT on admission**				
Typical appearance Atypical appearance Indeterminate appearance	146 (92.4) 2 (1.3) 10 (6.3)	94 (89.5) 2 (1.9) 9 (8.6)	52 (98.1) 0 (0.0) 1 (1.9)	0.151
ICU admission	35 (22.2)	16 (15.2)	19 (35.9)	0.003
ICU length of stay, days	7.0 (4.0–13.0)	8.5 (5.0–13.0)	5.0 (3.0–13.0)	0.443

NLCT, normal lung computed tomography; ALCT, abnormal lung computed tomography; BMI, body mass index; COPD, chronic obstructive pulmonary disease; CKD, chronic kidney disease; mMRC, modified medical research council score; LDH, lactate dehydrogenase; CT, computed tomography; ICU, intensive care unit. Data are presented as median (p25–p75) or *n* (%). *p*: normal vs. abnormal CT.

In the ALCT group, there were more men, smokers, and older people in the NLCT group (Table 1). ALCT group also had higher levels of D-dimer [1.4 (0.7–2.0) vs. 0.9 (0.6–1.3), *p* = 0.017] and ferritin [1,005.0 (564.0–2,350.7) vs. 691.0 (359.0–1,529.0), *p* = 0.022]. Of the total group, 22.2% of the patients were admitted to the ICU. These patients had lower PaO_2_/FiO_2_ (*p* < 0.001), higher levels of LDH (*p* = 0.036), ferritin (*p* = 0.008), leukocytes (*p* = 0.013), and more ALCT at 6 months after hospital discharge than those of the group that did not enter the ICU (*p* = 0.003) (Table 2). There were no significant differences in the total days of hospitalization between NLT and ALCT groups [7.0 (5.0–10.0) vs. 8.0 (6.0–12.0), *p* = 0.064].

**TABLE 2:** Baseline characteristics according to ICU admission (ICU and non-ICU patients).

	Total *N* = 158	No ICU *N* = 123	ICU *N* = 35	*p*
Age, years	55.0 (46.0–62.0)	53.0 (44.0–62.0)	58.0 (49.0–63.0)	0.208
Male, *n* (%)	91 (57.6)	65 (52.9)	26 (74.3)	0.024
BMI, kg/m^2^	26.5 (24.1–30.3)	26.7 (24.3–30.3)	25.8 (23.2–31.3)	0.459
Smoker/former smoker	41 (26)	29 (23.6)	12 (34.3)	0.202
Obesity	47 (29.8)	35 (28.5)	12 (34.3)	0.506
Asthma	7 (4.4)	6 (4.9)	1 (2.9)	0.608
COPD	4 (2.5)	4 (3.3)	0 (0.0)	0.280
Hypertension	51 (32.3)	37 (30.1)	14 (40.0)	0.268
Type 2 diabetes	19 (12.0)	12 (9.8)	7 (20)	0.100
CKD	3 (1.9)	3 (2.4)	0 (0.0)	0.351
Chest pain	30 (19.0)	27 (22.0)	3 (8.6)	0.075
Cough	122 (77.2)	94 (76.4)	28 (80.0)	0.656
mMRC 0	56 (35.4)	47 (38.2)	9 (25.7)	
mMRC ≥ 1	102 (64.6)	76 (61.8)	26 (74.3)	0.173
LDH, U/L	398 (304–497)	388 (295–484)	436 (266–549)	0.036
D dimer, mg/L	1.0 (0.6–1.6)	0.9 (0.6–1.5)	1.1 (0.7–1.9)	0.459
Ferritin ng/ml	801 (402–1,781)	838 (381–1,420)	1,120 (809–2,359)	0.008
White blood cell count, ×109/L	7,470 (5,490–9,935)	7,090 (5,230–9,660)	8,750 (6,520–12,000)	0.013
Lymphocyte count, ×109/L	1,040 (720–1,405)	1,050 (750–1,450)	870 (670–1,240)	0.136
PaO_2_/FiO_2_ ratio	245.4 (208.5–274.0)	249.0 (216.1–285.7)	207.5 (145.6–259.6)	< 0.001
**Chest CT on admission**				
Typical appearance Atypical appearance Indeterminate appearance	146 (92.4) 2 (1.3) 10 (6.3)	113 (91.9) 1 (0.8) 9 (7.3)	33 (94.3) 1 (2.9) 1 (2.9)	0.412
ALCT at 3 months	85 (53.8)	58 (47.2)	27 (77.1)	0.002
ALCT at 6 months	53 (33.5)	34 (27.6)	19 (54.3)	0.003

NLCT, normal lung computed tomography; ALCT, abnormal lung computed tomography; BMI, body mass index; COPD, chronic obstructive pulmonary disease; CKD, chronic kidney disease; mMRC, modified medical research council; LDH, lactate dehydrogenase; CT, computed tomography; ICU, intensive care unit. Data are presented as median (p25–p75) or *n* (%). *p*: normal vs. abnormal CT.

### Pulmonary function tests and symptoms

At the 3-month follow-up, FVC was similar in both groups, but a higher % of patients had FVC < 80% of predicted than in the ALCT group compared to NLCT (17.7 vs. 2.7%, *p* = 0.003). At the 6-month follow-up, there was no difference in FVC between ALCT and NLCT. Also, there were no differences in DLCO between the ALCT and NLCT groups at 3 or 6 months follow-up. At the 6-month follow-up, in both groups, there was an increase in FVC and DLCO, but 9.8% in NLCT and 14.3% in ALCT had FVC < 80% of predicted, and 14.6% in NLCT and 14.7% in NLCT had DLCO < of 70% of predicted.

**FIGURE 1:**
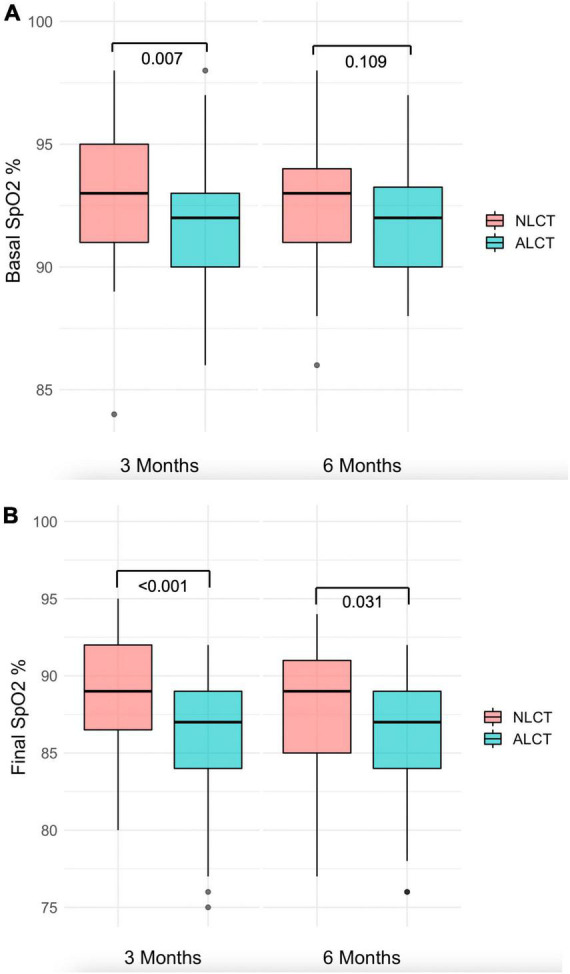
Basal and final SpO_2_ in 6MWT in NLCT and NLCT groups. **(A)** In the ALCT group, baseline SpO_2_ was lower than in NLCT both at 3 and 6 months of follow-up. **(B)** In the ALCT group, final SpO_2_ was lower than in NLCT at 6 months of follow-up. SpO_2_, oxygen saturación; 6MWT, six-minute walk test; NLCT, normal lung computed tomography; ALCT, abnormal lung computed tomography.

In the 6MWT there were no differences between the NLCT and ALCT groups in the meters walked at follow-up at 3 (*p* = 0.029) or 6 months (*p* = 0.667), although in both groups, the meters walked increased at 6 months (*p* < 0.001). The SpO_2_ at rest at the 3 months of follow-up was significantly lower in the ALCT group than in the NLCT group (*p* = 0.007) and also during exercise at the 3 (*p* < 0.001) and the 6 months of the follow (*p* = 0.031) (Figure 1). The PaO_2_/FiO_2_ ratio was lower in the ALCT group than in the NLCT at the 3-month follow-up (*p* = 0.016), with no difference at 6 months (*p* = 0.479) (Table 3).

**TABLE 3:** Pulmonary function test in follow-up at 3 and 6 months.

	Follow-up 3 months *N* = 158			Follow up 6 months *N* = 158				
	**NLCT** ***N* = 73**	**ALCT** ***N* = 85**	***p***	**NLCT** ***N* = 105**	**ALCT** ***N* = 53**	***p***	**NLCT** *	**ALCT** **
FVC,% of predicted	96.3 (91.0–107.4)	96.3 (85.3–111.0)	0.664	99.5 (91.1–108.9)	97.0 (84.7–109.9)	0.434	0.043	< 0.001
FVC, < 80% of predicted	2 (2.7)	15 (17.7)	0.003	12 (9.8)	5 (14.3)	0.445	0.563	0.008
FEV_1_, % of predicted	96.6 (88.1–107.3)	97.0 (86.9–107.5)	0.962	98.3 (90.0–107.8)	98.7 (86.1–111.3)	0.837	0.640	0.005
FEV_1_, < 80% of predicted	6 (8.2)	12 (14.1)	0.245	14 (11.4)	4 (11.4)	0.994	1.000	0.058
DLCO, % of predicted	90.7 (80.9–100.7)	86.3 (73.2–101.1)	0.156	93.1 (81.1–101.4)	88.5 (75.9–97.5)	0.155	0.008	0.001
DLCO, < 70% of predicted	7 (9.6)	16 (19.1)	0.095	18 (14.6)	5 (14.7)	0.992	0.102	0.179
**6MWT**								
Meters	582.5 (531–636.5)	559.0 (494–614)	0.229	598.0 (527.0–649.0)	587.0 (526.0–646.0)	0.667	< 0.001	< 0.001
Basal SpO_2_, %	93.0 (91.0–95.0)	92.0 (90.0–93.0)	0.007	93.0 (91.0–94.0)	92.0 (90.0–93.5)	0.109	0.899	0.894
Final SpO_2_, %	89.0 (86.0–92.0)	87.0 (84.0–89.0)	< 0.001	89.0 (85.0–91.0)	87.0 (84.0–89.0)	0.031	0.154	0.266
PaO_2_/FiO_2_ ratio	310.5 (288.1–338.1)	295.7 (275.7–316.9)	0.016	300.5 (282.9–316.2)	295.0 (275.0–319.0)	0.479	0.315	0.370

NLCT, normal lung computed tomography; ALCT, abnormal lung computed tomography; FVC, forced vital capacity; FEV_1_, forced expiratory volume in the first 1 s; DLCO, carbon monoxide diffusion capacity; 6MWT, six-minute walk test; SpO_2_, oxygen saturation; PaO_2_, partial pressure of arterial oxygen; FiO_2_, fraction of inspired oxygen. Data are presented as median (p25–p75), or *n* (%). *p*: NLCT vs. ALCT at 3 and 6 months. *p: differences between 3 and 6 months in NLCT group; **p: differences between 3 and 6 months in ALCT.

There were no differences between groups in the presence of cough, chest pain, or dyspnea on admission to hospitalization or at 3-month follow-up. At the 6-month follow-up, patients with ALCT had more dyspnea (mMRC ≥ 1) than those with NLCT (32.1 vs. 15.8%; *p* = 0.021).

### CT scan findings

**FIGURE 2:**
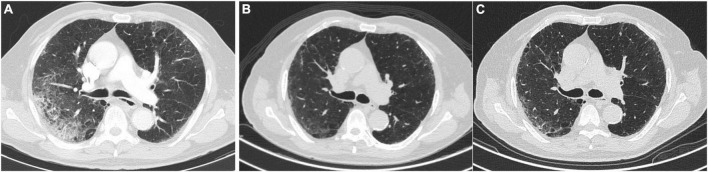
Changes in the CT scan in a patient with COVID pneumonia. **(A)** Upon admission. **(B)** At 3 months of follow-up. **(C)** At six-month follow-up.

On admission, 92.4% of the patients had CT scans typical findings of SARS-CoV-2 infection, with no differences between the NLCT and ALCT groups (*p* = 0.412). In the follow-up 6 months after hospital discharge, the most frequent pattern of alteration in the ALCT group was the GGO (31.7%), followed by the reticular pattern in 2.5%, and no patient presented findings of traction bronchiectasis or honeycomb (Figure 2).

In the multivariate analysis, the variables associated with the persistence of infiltrates on CT at 6 months were older age, male sex, stay in the ICU, and the typical pattern on admission CT, test de Hosmer–Lemeshow *p* = 0.816 and the AUROC curve was 0.768 (Table 4).

**TABLE 4:** Multivariate analysis of risk factors associated with ALCT in follow-up at 6 months.

	Univariate		Multivariate	
	**OR (95% CI)**	***p***	**OR (95% CI)**	***p***
Age, years	1.056 (1.024–1.088)	< 0.001	1.068 (1.031–1.107)	< 0.001
Sex, male	2.485 (1.222–5.053)	0.012	2.692 (1.223–5.925)	0.014
CT scan typical appearance	6.085 (0.764–48.462)	0.088	9.738 (1.108–85.564)	0.040
ICU hospitalization	3.108 (1.434–6.737)	0.004	2.516 (1.077–5.873)	0.033

ALCT, abnormal lung computed tomography; ICU, intensive care unit. Test de Hosmer–Lemeshow *p* = 0.816.

## Discussion

In this cohort of patients with moderate to severe COVID-19 disease, evaluated at a fourth-level hospital in the city of Bogotá, it was shown that 33.5% had ALCT at 6 months of follow-up. Although there were no differences in FVC and DLCO between ALCT and NLCT, those with ALCT had more dyspnea and lower SpO_2_ during exercise than patients with NLCT. The factors associated with the persistence of infiltrates in the CT scan at 6 months were older age, male gender, presence in the ICU, and the typical pattern in the CT scan at hospital admission.

In our cohort, the main abnormality on the CT scan at 6 months of follow-up was the presence of GGO. This finding has been described as occurring in 80% of patients with COVID-19 at 2–3 months after admission to the hospital, and in 40% at 6–7 months of follow-up (8). In a study that included 114 survivors of severe COVID-19, the lesions found at 6 months of follow-up were GGO, interstitial thickening, and fibrotic lesions (traction bronchiectasis and parenchymal bands) in 27, 35, and 9%, respectively (20). Another study in 83 patients in the city of Wuhan, showed that the GGO was the main finding in the CT scan at 3 months of follow-up in 78% of the patients, and at 6 months in 46%, without complete resolution at 9 months of follow-up (9). Recently, a cohort from Spain of 284 patients with a 1-year follow-up reported alterations in tomography in 123 patients, with the presence of GGO in 47%, reticulation in 19%, and the presence of parenchymal bands in 22% (21).

The risk factors for the presence of ALCT at 6 months of follow-up in our cohort were older age, male sex, stay in the ICU, and the presence of a typical pattern in the chest CT on hospital admission. These findings are similar to those described in the 1-year follow-up in the COVID-FIBROTIC study team cohort from Spain, where they found that initial radiological compromise on admission was associated with the persistence of persistent tomographic lesions (21).

Another of the Wuhan cohorts, with follow-up at 1 year, described that ICU admission was associated with the presence of GGO at 1 year of follow-up (22). In the 7-month follow-up of tomographic sequelae in another cohort from China, the risk factors for the presence of these ALCTs were older age, longer hospital stay, and the need for ICU hospitalization (23), similar to that described in our cohort. Although there are few works that describe the presence of symptoms after SARS-CoV-2 infection differentiated between men and women, systematic reviews describe more severe diseases in men and greater residual respiratory symptoms in women (24), similar to our results.

The characteristics of populations infected with SARS-CoV-2 vary according to their geographic distribution and epidemiological characteristics at the time of infection. In our cohort, there were more men, and among the comorbidities that were found the most were smoking, arterial hypertension, obesity, and type 2 diabetes, as previously described in cohorts from Latin America, Wuhan, Italy, and New York (25–27). In Latin America, different risk factors for COVID-19 infection that are associated with the severity of the disease have been reported (28). In Colombia, it has been described that age, male gender, and the presence of comorbidities are associated with admission to the ICU (29), and in Bogotá, older age, lower PaO_2_/FiO_2_ ratio, and higher LDH at admission were associated with higher mortality (13). Similarly, the patients in our cohort that were admitted to ICU were more frequently male and with lower PaO_2_/FiO_2_ and higher levels of LDH.

Although smoking has also been associated with tomographic and functional abnormalities and greater severity of symptoms in patients with COVID-19 (21, 22), in our study it was not associated with ALCT in the multivariate analysis, similar to that described in other studies with follow-ups of 6 and 12 months (8, 23).

In our cohort, it occurred in less than 25%, and in the multivariate analysis, it was not associated with ALCT, similar to that described in follow-up cohorts of more than 6 and 12 months.

In the follow-up of lung function in SARS-CoV-2, restrictive and obstructive alterations in spirometry and a decrease in DLCO have been found (30). Several studies have shown that the reduction of DLCO in combination with restrictive patterns was the most frequent parameter in the follow-up of these patients (31, 32). At 12 months post-COVID, Huang et al. found that spirometry values were normal, and there was a decrease in DLCO (< 80% of predicted) in 23% of patients with moderate disease and 31% with severe disease (22). In another study with a 6-month follow-up, the reduction in DLCO occurred between 22 and 56% of the patients and was associated with the severity of COVID-19 and the need for hospitalization (8). Strikingly in our cohort, there were no differences between the ALCT and NLCT groups in FVC or DLCO at 6-month follow-up, but about 10% of patients had FVC < 80% predicted, and 15% of patients had DLCO < 70% of predicted.

It has been described that patients infected by SARS-CoV-2 have less exercise capacity after 6 months of infection, compared to the population without infection (33). In 2005, Hui et al. followed up 110 SARS survivors, noting that decreased DLCO was the most frequent finding of impaired lung function, and that those who had been admitted to the ICU walked fewer meters in the 6MWT (5). At sea level, in patients with SARS-CoV-2 pneumonia, the average number of meters walked reported in the 6MWT at 3 months was 539 ± 102.8 m, which increased in the year of follow-up to 556 ± 92 m (34). In Spain, in a prospective study carried out at sea level, the average number of meters walked at 2 months of follow-up was 524 m, at 6 months 521 m and at 12 months 519 m (21). In Latin America, a Mexican group located at sea level in the Yucatán Peninsula described that patients with mild to severe COVID-19 disease followed by persistent dyspnea walked an average of 493 ± 7 m (35). In our cohort of moderate to severe COVID survivors, there were no differences between the ALCT and NLCT groups in meters walked at 3 or 6 month follow-up, with values higher than those reported in previous studies (21, 33–35).

Despite more meters walked, the saturation values during exercise in our cohort were lower than those reported at sea level (30, 31, 36), even in severe pneumonia cohorts followed up only 2 months after symptom onset with average saturations greater than 97% (36). These differences in saturation can be explained by altitude. Due to Bogotá’s location at 2,640 m above sea level, PaO_2_ is around 60 mmHg and SaO_2_ is 90% in normal patients, with significant desaturations during exercise in patients with interstitial lung disease (11). Despite these lower saturations at altitude, different studies suggest that this does not represent a negative impact on the mortality of patients with COVID-19 residing at high altitudes (37).

At the 6-month follow-up, dyspnea was the main symptom in patients with ALCT, which occurred significantly more frequently than in the NLCT group. These findings are consistent with the new definitions of the long-term effects of COVID-19 or Long-COVID-19, in which dyspnea occurs in up to 61% of patients suspected of having this syndrome (38). In addition, the presence of dyspnea has been reported in more than half of the patients during physical activity, even without having abnormalities in the pulmonary function tests (39), similar to the results of our cohort (40).

In this study, with a significant number of patients, we show the medium-term behavior of patients with moderate and severe COVID-19 pneumonia at high altitude. We emphasize that clinical follow-up was achieved up to 6 months after hospitalization with symptoms, functional evaluation with spirometry, DLCO, 6MWT, and CT scan. Among the limitations of the study, we highlight that it was carried out in a single center in the city of Bogotá, and the patients included were younger and had fewer comorbidities than those included in other cohorts with COVID-19. The study was carried out before vaccination against COVID and during the first and second epidemiological peaks in the country, which could determine that the population had a more severe disease with greater alterations in the follow-up computed tomography. We also did not record the type of ventilatory support in patients admitted to the ICU and the number of patients with pulmonary embolism as a complication of COVID-19 during hospitalization, although none of the patients had anticoagulant treatment at post-hospitalization follow-up visits. Another point of improvement was that we did not evaluate the fatigue symptom since we focused only on the presence of cough, chest pain and dyspnea. Finally, the 6-month evaluation period, although longer than most studies, is shorter than other studies with post-COVID follow-up of up to 1 year.

## Conclusion

Our study, conducted in a high-altitude city, showed that one-third of patients hospitalized for moderate and severe COVID have persistent chest tomography abnormalities 6 months after discharge, with lower exercise saturation and more dyspnea than those without persistent pulmonary infiltrates.

## Data availability statement

The original contributions presented in this study are included in this article/supplementary material, further inquiries can be directed to the corresponding author.

## Ethics statement

The studies involving human participants were reviewed and approved by the Ethics Committee of the Fundación Neumológica Colombiana (approval number 202007-25702). The patients/participants provided their written informed consent to participate in this study. Written informed consent was obtained from the individual(s) for the publication of any potentially identifiable images or data included in this article.

## Author contributions

ER-A, MG-G, CT-D, CP, WR, MC, AM, and PP-T: conception and design. NP and ER-A: analysis and interpretation. MG-G, LG-C, ER-A, MG-G, CT-D, AA-M, and AC: drafting the manuscript for important intellectual content. All authors contributed to the article and approved the submitted version.
